# Highlights on molecular targets for radiosensitization of breast cancer cells: Current research status and prospects

**DOI:** 10.1002/cam4.1588

**Published:** 2018-06-01

**Authors:** Zhi‐Rui Zhou, Zhao‐Zhi Yang, Xiao‐Li Yu, Xiao‐Mao Guo

**Affiliations:** ^1^ Department of Radiation Oncology Fudan University Shanghai Cancer Center Shanghai China; ^2^ Department of Oncology Shanghai Medical College Fudan University Shanghai China

**Keywords:** breast cancer, molecular targets, radiation sensitization, radiotherapy

## Abstract

In the past, searching for effective radiotherapy sensitization molecular targets and improving the radiation sensitivity of malignant tumors was the hot topic for the oncologists, but with little achievements. We will summarize the research results about breast cancer irradiation sensitization molecular targets over the past two decades; we mainly focus on the following aspects: DNA damage repair and radiation sensitization, cell cycle regulation and radiation sensitization, cell autophagy regulation and radiation sensitization, and radiation sensitivity prediction and breast cancer radiotherapy scheme making. And based on this summary, we will put forward some of our viewpoints.

## 1 INTRODUCTION

The past two decades have witnessed the arduous efforts of radiation oncologists in searching for effective radiosensitization targets and improving radiosensitivity of malignant tumors. Although previous literature has described many potential molecular targets for radiosensitization,^1^ few targets can be translated into radiosensitizers that can be used in clinical settings. Studies on the molecular targets for radiosensitization of breast cancer have been published since the 1990s. In 1994, Wollman et al^2^ found that treatment of breast cancer MCF‐7 cells with epidermal growth factor (EGF) before irradiation could stimulate cell proliferation and increase their radiation resistance. In 1996, Sakakura et al^3^ reported that Bcl‐2 overexpression could increase the radiosensitivity of breast cancer MCF‐7 cells by increasing cell apoptosis. In 1997, Turner et al^4^ reported that insulin‐like growth factor‐I (IGF‐I) receptor overexpression mediated the radiation resistance of breast cancer cells and led to tumor recurrence after breast radiotherapy after breast‐conserving surgery (BCS). The inhibition of estrogen level by tamoxifen may increase the radiation resistance of breast cancer, whereas estrogen treatment may heighten the radiosensitivity of breast cancer cells; paradoxically, the use of estrogen inhibitors is common in breast cancer patients.^5^ With the rapid development of molecular biology in the 21st century, research on the potential molecular targets for the radiosensitization of breast cancer has become a hot topic. In this article, we will review the studies on molecular targets for the radiosensitization of breast cancer in the past 20 years, with an attempt to shed a light on its future developments.

## 2 CURRENT RESEARCHES

### 2.1 DNA damage repair and radiosensitization

One of the determinants of radiosensitivity is the efficiency of DNA double‐strand damage repair.^6^ The DNA double‐strand breaks can be repaired in two manners: homologous recombination (HR) and nonhomologous end‐joining (NHEJ).^7^ These two repair modes coordinate with each other, jointly maintaining the stability of organisms’ genomes. HR occurs mainly in the late S phase and the G2 phase, during which Rad51/52/54 complex, BRCA1/BRCA2, XRCC2, and XRCC3 proteins are involved in this repair process. Unlike HR, NHEJ requires no homologous template repair; rather, it occurs mainly in the G1 phase of a cell cycle, during which proteins including Ku70/KuS0, DNA‐PK, XRCC4, and Ligase 4 participate in this process.^1^

Radiosensitization of malignant tumors by regulating molecules associated with the repair of DNA damage has long been a hot research topic. A consistent viewpoint is that it inhibits molecules associated with cell DNA damage repair after irradiation exposure which can increase the cell’s sensitivity to the rays; the conclusions have been verified in multiple DNA damage related to molecules.^1^, ^8^, ^9^, ^10^, ^11^, ^12^, ^13^, ^14^, ^15^, ^16^, ^17^, ^18^, ^19^, ^20^, ^21^, ^22^, ^23^, ^24^, ^25^, ^26^, ^27^, ^28^, ^29^, ^30^, ^31^, ^32^ Wang et al^33^ reported that PARP inhibitor, MK‐4827, radiosensitized human lung and breast cancer xenografts regardless of their p53 status show high potential to improve the efficacy of radiotherapy in phase I clinical trial. Cruz et al reported that BRCA1 and BRCA2 (BRCA1/2)‐deficient tumors display impaired homologous recombination repair (HRR) and enhanced sensitivity to DNA damaging agents or to PARP inhibitors (PARPi). Numerous mechanisms of PARPi resistance have been described; PARPi resistance could be reverted upon combination of a PARPi with an ATM inhibitor in patient‐derived tumor xenografts.^34^ Table 1 lists the relationships between the molecular targets associated with DNA damage repair and the radiosensitivity of breast cancer cells.

**Table 1:** A list of genes associated with radiosensitivity of breast cancer

Gene name	Biological function	Cell response to radiation
Ku70/80 (XRCC6/5)	Nonhomologous end‐joining (NHEJ)	Radiosensitive when deficient or decreased^1^, ^8^, ^9^, ^10^
DNA‐PKcs (PRKDC)	NHEJ	Radiosensitive when deficient or decreased^1^, ^11^, ^12^
XRCC1/XRCC2/XRCC3/XRCC4	NHEJ	Radiosensitive when deficient or decreased^1^, ^13^, ^14^, ^15^, ^16^
DNA Ligase	NHEJ	Radiosensitive when deficient or decreased^1^, ^17^, ^18^, ^19^
RAD51	Homologous recombination (HR)	Radiosensitive when deficient or decreased^20^, ^21^, ^22^
RAD54	HR	Radiosensitive when the expression level is deficient or decreased^1^, ^24^, ^25^, ^26^
BRCA1	HR, cell cycle, apoptosis, etc.	Radiosensitive when deficient or decreased^27^, ^28^
BRCA2	HR	Radiosensitive when deficient or decreased^12^, ^23^, ^35^
ATM	Cell signal transduction in DNA damage	Radiosensitive when deficient or decreased^29^, ^30^
PARP‐1	DNA damage repair	Radiosensitive when deficient or inhibited^31^, ^32^
PTEN	Encoding phosphorylate	Probably radiosensitive when deficient or inhibited. The conclusions in different literatures are inconsistent^1^, ^36^, ^37^, ^38^, ^39^
TP53	Transcription, cell cycle, apoptosis	Probably radiosensitive when deficient or inhibited. The conclusions in different literatures are inconsistent^40^, ^41^, ^42^, ^43^
Cyclin D1 (CCND1)	G1 cell cycle regulation	Radioresistant when overexpression^38^, ^44^, ^45^, ^46^
WEE1	G2 cell cycle regulation	Probably radiosensitive when inhibited^20^, ^47^
CHEK1	Cell cycle regulation	Radiosensitive when inhibited^29^, ^47^, ^48^, ^49^, ^50^
TP21	Cell cycle regulation	Radiosensitive when deficient or inhibited^17^, ^51^
ERBB2	Growth factor	Radioresistant when overexpression^52^
ATGs	Cellular autophagy	Radiosensitive when inhibited^48^, ^53^, ^54^, ^55^, ^56^, ^57^, ^58^
AR	Androgen receptor	Radioresistant when deficient or inhibited^59^, ^60^, ^61^, ^62^
JUN	Proto‐oncogene, transcription factor	Radiosensitive when deficient or inhibited^59^, ^60^, ^61^, ^62^
STAT1	Signal transduction, transcription activators	Radiosensitive when deficient or inhibited^59^, ^60^, ^61^, ^62^
PKC	Serine‐ and threonine‐specific protein kinases	Radioresistant when deficient or inhibited^59^, ^60^, ^61^, ^62^
RelA (TP65)	Nuclear transcription factor	Radioresistant when deficient or inhibited^59^, ^60^, ^61^, ^62^
c‐ABL	Non‐receptor tyrosine kinase	Radiosensitive when deficient or inhibited^59^, ^60^, ^61^, ^62^
SUMO‐1	Posttranslational regulation	Radioresistant when deficient or inhibited^59^, ^60^, ^61^, ^62^
CDK1 (TP34)	Cell cycle regulation	Radioresistant when deficient or inhibited^59^, ^60^, ^61^, ^62^
HDAC1	Histone deacetylation	Radioresistant when deficient or inhibited^59^, ^60^, ^61^, ^62^
IRF1	Activating transcription factor	Radioresistant when deficient or inhibited^59^, ^60^, ^61^, ^62^

### 2.2 Cell cycle regulation and radiosensitization

Radiation‐induced DNA damage may induce G1/G2 arrest; as a result, the damaged cells have enough time to repair the damage. Based on this theory, targeted suppression of the G1 or G2 arrest may synergize the killing effect of radiation. Many molecular targets such as TP53, TP21, CDK1, CHEK1, and WEE1 have been investigated. Table 1 lists some of the cell cycle regulation genes that may be associated with radiosensitization. While the first three gene‐specific targeting inhibitors have not been reported, there is more literature on the selective inhibitors of the latter two targets.

Among them, the cell cycle checkpoint kinase CHEK1 is one of the important proteins that negatively regulates the cell cycle. It can cause cell cycle arrest once being activated. Up to now, many specific small molecular inhibitors have been developed for in vitro and in vivo experiments.^63^, ^64^ CHEK1 can be activated when ATM/ATR kinase detects DNA double‐strand breaks or large‐scale damage to single DNA strands. Once CHEK1 is activated, its phosphorylated product can inhibit the CDC25 phosphorylation, which is a prerequisite to the smooth progression of the cyclin‐dependent kinases (CDKs) and cell cycles. Inhibition of CHEK1 can lead to the phosphorylation of CDC25, which activates CDK1/2 to make the cells successfully progress through the G2/M phases. When the damaged cells prematurely pass through the cell cycle, the DNA damage may have not been repaired. The accumulation of DNA damages can cause fatal cell damage or mitotic catastrophe, which is obviously unfavorable for cells.^65^ A variety of inhibitors of this target have been developed and commercialized. Among them, MK‐8776 is a small molecular inhibitor that selectively inhibits CHEK1 protein.^63^ Previous studies have shown that MK‐8776 could synergistically increase the toxicities of chemotherapeutic agents such as Hydroxyurea and Gemcitabine to tumor cells in vitro and in vivo but did not increase their toxicities to normal tissue cells.^63^, ^66^, ^67^ Our study showed MK‐8776 could synergize the radiosensitization of radiation in triple‐negative breast cancer cells.^48^

In addition, WEE1 is also one of the key enzymes that regulates the G2 checkpoint. It can inhibit the Cdc2 kinase by phosphorylation of Cdc2 at the Tyr15, leading to the inactivation of the Cdc2/cyclin B kinase‐proteins (an important regulator of G2/M phase); as a result, the cells are arrested in G2 phase and cannot enter M phase.^68^ At present, a variety of WEE1 inhibitors including PD0166285, PD0407824, and MK‐1775 have been developed. Among them, MK‐1775 is a highly selective small molecule inhibitor. Studies have confirmed that MK‐1775 can effectively regulate the sensitivity of cells to DNA‐damaging chemotherapy drugs or radiation. Hirai et al^69^ found that MK‐1775 could selectively inhibit WEE1 kinase and significantly increased the cytotoxic effects of gemcitabine, carboplatin, and cisplatin on solid tumor cells. In a study on non‐small cell lung cancer, Bridges et al^70^ found that MK‐1775 could significantly increase the radiosensitivity of lung cancer cells to radiation by inhibiting G2 phase arrest. Sarcar et al^71^ concluded that MK‐1775 significantly increased the radiosensitivity of glioblastoma. Murrow et al^72^ and Iorns et al^73^ found that the inhibition of WEE1 kinase could lower the proliferation of breast cancer cells, reduce phase G2 arrest, increase cellular γH2A.X level, and increase the number of apoptotic cells. Garimella et al^74^ discovered that MK‐1775 could increase the apoptosis of breast cancer cells by synergizing with TNF‐related apoptosis‐inducing ligand (TRAIL). Clearly, cell cycle regulation is a valuable research direction for radiosensitization of breast cancer.

### 2.3 Autophagy regulation and radiosensitization

Autophagy was a new discovery of cell death in the field of biology in the 1970s. Many previous studies have focused on the role of autophagy in pathogenesis and disease treatment. While autophagy insufficiency may be associated with the occurrence of the disease, increased autophagy may also affect the efficacy of clinical treatment.^75^, ^76^, ^77^, ^78^, ^79^, ^80^, ^81^ Autophagy has multifaceted roles in the occurrence and treatment of tumors.^82^, ^83^, ^84^ On the one hand, the knockout of autophagy‐associated gene (ATG) or the inhibition of autophagy by drugs can increase spontaneous tumors in animal models; on the other hand, inhibition of autophagy in tumor cells increases their sensitivity to chemotherapy and radiotherapy.^81^

Studies have shown that irradiation can lead to obvious autophagy in nasopharyngeal carcinoma cell line CNE‐2. The radiosensitivity of nasopharyngeal carcinoma cells can be significantly increased after the inhibition of autophagy using chemical inhibitors.^81^ In this phenomenon, DNA damage repair‐associated protein PARP‐1 is involved in regulating the radiation‐induced autophagy, and inhibiting autophagy can increase the lethal effect of radiation on cells.^81^ Our research also shows that the selective inhibitor of CHEK1 can inhibit autophagy in triple‐negative breast cancer cells and thereby increase the radiosensitivity of triple‐negative breast cancer.^48^ Kim et al^56^ found that endoplasmic reticulum stress in the Caspase3/7‐deficient breast cancer cells may be a potential mechanism of radiation‐induced autophagy, which may serve as a potential radiosensitization strategy to maximize the killing efficiency of radiotherapy on breast cancer cells. Chaachouay et al^58^ found that, compared with the radiosensitive HBL‐100 breast cancer cells, the radio‐resistant MDA‐MB‐231 breast cancer cells have a remarkably higher level of autophagy after irradiation, and thus autophagy played a key role in protecting breast cancer cells against radiation. Han et al found that autophagy inhibitors increased the sensitivity of MDA‐MB‐231 breast cancer cells to radiation by inhibiting the activation of TAK1 after irradiation, suggesting that regulating TAK1 may be an effective way in treating radiation‐resistant breast cancer.^53^ Sun et al^54^ have found that MiR‐200c inhibited autophagy and enhanced radiosensitivity in breast cancer cells by targeting UBQLN1. In summary, inhibition of cell autophagy can increase the radiosensitivity of breast cancer cells.

### 2.4 Radiosensitivity prediction and radiotherapy schemes making

Encouragingly, the research team from the H. Lee Moffitt Cancer Center of the University of Florida College of Medicine, led by Dr. Javier F. Torres‐Roca, has published a series of articles in the past decade, showing us the way to establish prediction models for malignancy radiosensitivity and the clinical values of these models.^59^, ^60^, ^61^, ^62^, ^85^, ^86^, ^87^, ^88^, ^89^ Firstly, they carried out gene expression microarray analyses in 48 different cell lines of 9 different malignant tumors including breast cancer, colorectal cancer, nervous system tumor, melanoma, lung cancer, ovarian cancer, prostate cancer, renal cell carcinoma, and blood disease. Based on the inherent differences in the radiosensitivity among these cell lines, 10 differentially expressed genes related to cell radiation sensitivity were identified. The names and biological functions of these genes have been listed in Table 1. The authors have constructed a linear regression model based on the expression levels of these 10 genes and calculated the radiosensitivity index (RSI) to predict the radiosensitivity of the cancer cells. RSI is expressed as the surviving fraction of cells after 2 Gy of radiation (SF2). The linear regression model is constructed as follows: [RSI = (−0.0098009 × AR) + (0.0128283 × c‐Jun) + (0.0254552 × STAT1) − (0.0017589 × PKC) − (0.0038171 × RelA) + (0.1070213 × cABL) − (0.0002509 × SUMO1) − (0.0092431 × CDK1) − (0.0204469 × HDAC1) − (0.0441683 × IRF1). *Formula 1*]. High RSI score suggests the presence of radiation resistance, whereas low RSI score represents high sensitivity to radiation. Using systematic biological methods, the authors found that a signal network mutually regulated by these 10 genes determined the radiosensitivity of the cells.^59^, ^60^ The authors further validated their findings in different cohorts from multiple centers. In different cancers (eg breast cancer, colorectal cancer, head and neck squamous cell carcinoma, esophageal cancer, pancreatic cancer, and colorectal cancer) treated with radiotherapy, it was confirmed that RSI was associated with the prognosis: patients with lower RSI score (radiotherapy‐sensitive) had better prognosis, whereas patients with higher RSI score (radiotherapy‐resistant) had poorer prognosis. In addition, RSI has good predictive value for patients receiving radiotherapy and can be universally applied in the prediction of different cancer types.^61^, ^62^, ^85^, ^86^, ^87^, ^88^, ^89^

In the clinical practice of radiotherapy, the effectiveness of radiotherapy is not only related to the radiosensitivity of tumor cells but also associated with the total dose and the fraction numbers of radiotherapy. With an attempt to take factors associated with radiobiology and radiation physics into consideration, the authors combined the widely used linear quadratic equation (L‐Q formula) with the radiosensitivity index (RSI) and proposed the concept of genomic‐adjusted radiation dose (GARD). Thus, the concept of GARD is a new mathematical model that integrates the expression information of individual genes, considers the dose and fraction number of radiotherapy, and predicts the prognosis of patients receiving radiotherapy. In this model, a higher GARD value suggests a better prognosis for patients receiving radiotherapy. The formula is as follows: [*E* = nd(α + β*d*). *Formula 2*]. “*E*” represents the GARD value, in which “n” and “*d*” denote the number of fractions and the single irradiation dose, respectively; the “α*”* value denotes the radiosensitivity index (RSI), whereas “β*”* is a constant (0.05/Gy^2^).

The researchers also explored the relationship between the GARD value and the radiotherapy dose. Based on the radiotherapy doses, the tumor patients were divided into low‐dose (45 Gy/25fx), intermediate‐dose (60 Gy/30fx), and high‐dose (70 Gy/35‐40fx) groups. Gene expressions in tumor tissue samples were detected in 8271 patients; the GARD values were calculated and sorted. It was observed that the GARD value was not only related to the dose of radiotherapy; rather, the GARD value could be high in the low‐dose group and could be low in the high‐dose group. The range of GARD value was 3.03‐56.34 in the low‐dose group, 1.66‐122.38 in the intermediate‐dose group, and 9.73‐172.4 in the high‐dose group. Furthermore, the researchers also studied the relationship between the GARD value and the sensitivity of tumor radiotherapy. The distribution of GARD values differed for patients with different tumors receiving the same dose of radiotherapy. For example, the GARD value of patients with cervical cancer or head neck oropharyngeal tumor receiving 70 Gy was higher than those of patients with other tumors receiving the same dose of irradiation, which was consistent with the sensitivity of cervical cancer, head neck, and pharynx tumor to radiotherapy. Among tumors treated with the same dose of 60 Gy, the GARD values of glioma and sarcoma were lower than those of other tumors, which was consistent with the radiation resistance of glioma and sarcoma in clinical settings. Finally, the authors concluded that the GARD value was associated with the prognosis. The GARD values differ in different individuals and in different tumor types, which suggests the response to treatment differs among different individuals. Validation studies in five different tumor cohorts (including two breast cancer cohorts, one glioblastoma cohort, one lung cancer cohort, and one pancreatic cancer cohort) showed that the GARD values were independently correlated with clinical prognosis. For 263 breast cancer patients in the Erasmus database, they were grouped according to the GARD values. Patients with high GARD values had better radiotherapy response than those with low GARD values, along with longer 5‐y metastasis‐free survival; Cox regression analysis showed that the GARD value was a better predictive factor than RSI alone or biological effective dose (BED).^88^

## 3 CHALLENGES AND PROSPECTS

We believe that research on the radiosensitization of malignant tumors should include two aspects: firstly, to find potential targets for radiosensitization and develop new radiosensitizing drugs; and second, to find the molecular markers for the accurate judgment and prediction of radiosensitivity of malignant tumors.^90^ In the past, most of the studies focused on the former, but with little achievements. In addition to breast cancer, this was also true for research on the radiosensitization of other solid tumors. Few molecular targets found in basic research have been translated into clinically feasible radiosensitizing drugs (nontoxic for cells when used alone and has synergistic effect when used in combination with radiation). For such a situation, radiation oncologists may be frustrated. Why? Toxicologically, the toxicity of a substance depends on its intake dosage and the subjects’ sensitivity to it. In other words, when the dosage is high enough, any substance can be toxic for a subject. In this sense, it can be extremely difficult to find a radiosensitizer in pure sense. This means that the previous radiosensitization studies might be in a questionable direction. We should give up the effort to find pure radiation sensitizing drugs in a timely manner and should focus on the combined effects of drugs and irradiation. If the combination of a drug and irradiation is more effective than a single method, such drugs will be valuable, and this is a more pragmatic approach. Furthermore, GARD study is a successful example of the translation of basic radiobiological findings into clinical applications,^88^ and it provides us with new insights and new paradigms in the translational studies on radiosensitization: it is of more practical significance to find molecular markers that can be used to judge and predict the radiosensitivity of malignant tumors. By establishing models for predicting the radiosensitivity of malignant tumors with these markers, we can judge the possible response to radiotherapy and chemotherapy before a cancer patient receives these treatments and establish treatment schemes more accurately, which are also real‐world issues to be solved in precision medicine.

Most of the previous studies have explored radiosensitivity from the perspective of a single molecular target. In fact, the biological function of an organism is regulated by complex cell signaling networks. These single‐faceted studies could not offer a comprehensive view. However, this does not mean these studies were useless; in fact, they paved the way for new discoveries. Today, with the rapid development of genomics and other research techniques, it is no longer difficult to screen and verify high‐throughput radiosensitization targets. In the future, we should search for targets for radiosensitization on the “lines” or “surfaces” of the signaling networks, followed by the use of high‐throughput verification to achieve the verification of multiple targets. Eschrich et al^60^ has provided us with a good research paradigm in investigating radiosensitivity‐associated molecular networks using systematic biological methods. In addition, previous studies have often been limited to the search for radiation sensitization targets in one single cancer type. Of course, there was nothing wrong. We always learn a new thing from a “point”. However, when we accumulate enough knowledge from multiple “points”, we should understand such a thing from a more macro‐perspective. Sometimes, a single molecular target is related to either radiosensitivity or radioresistance in different cancers. This may be explained by the fact, as mentioned above, that the functions of organisms are regulated by signaling networks. However, another explanation is that such a molecular target actually plays different roles in different cancer types. Such “dual‐faceted” molecular targets may not be good potential radiosensitization targets. It is assumed that there must be some more broad‐spectrum targets for radiosensitization: they are functionally stable and can achieve radiosensitization in a variety of tumors.

Previous studies were only carried out on a cellular or animal level, and few of them have been translated/validated in clinical samples. It is well known that in vitro or animal experiments often cannot tell the true story of a human body. Of course, this is not a unique phenomenon. The success rate of translating research into practice remains low, particularly the research on radiosensitization targets. In the past few decades, radiation oncologists and radiologists have successfully found many potential radiosensitization targets. Future studies should focus on the clinical translation of in vitro radiosensitizing targets, so that more basic experimental results can be applied in predicting radiosensitivity and developing radiosensitizing drugs for patients with malignant tumors. Ultimately, these achievements will benefit patients and help conquer cancers.

## CONFLICT OF INTEREST

The authors declare no conflicts of interest.
